# *Onthodiplogaster japonica* n. gen., n. sp. (Rhabditida: Diplogastridae) isolated from *Onthophagus* sp. (Coleoptera: Scarabaeidae) from Japan

**DOI:** 10.1038/s41598-023-33586-1

**Published:** 2023-04-20

**Authors:** Natsumi Kanzaki, Yuya Ikeda, Ryoji Shinya

**Affiliations:** 1 Kansai Research Center, Forestry and Forest Products Research Institute, 68 Nagaikyutaroh, Momoyama, Fushimi, Kyoto, 612-0855 Japan; 2 School of Agriculture, Meiji University, Kawasaki, Kanagawa 214-8571 Japan

**Keywords:** Ecology, Evolution, Zoology

## Abstract

A diplogastrid nematode was isolated from a dung beetle, *Onthophagus* sp., collected from a rotten mushroom in Kyoto, Japan. The species is characterised by its cheilostomatal shape, separated into 12 narrow plates (rugae), deep stegostom, large ellipsoidal amphids, conical female tail and characteristic *receptaculum seminis* in the female. Based on its phylogenetic status and stomatal composition, the species is typologically similar to two other diplogastrid genera, *Neodiplogaster* and *Mononchoides*. The species can be distinguished from these two genera by the size and shape of the amphid (small pore in *Neodiplogaster*), female tail shape (long and filiform in *Mononchoides*) and presence of *receptaculum seminis* (absence in the two nominal genera), and is described as a monotypic member of a new genus, *Onthodiplogaster japonica* n. gen., n. sp. Observation of feeding behaviour suggested that *O. japonica* n. gen., n. sp. does not show clear stomatal dimorphism or polymorphism, which is found in its close relatives, but the species can feed on nematodes (predation), fungi and bacteria. This monomorphic omnivory possibly represents its habitat of dung and other rotten materials, where the environment is biologically divergent, and its condition changes rapidly.

## Introduction

The nematode family Diplogastridae Micoletzky is a highly divergent group with respect to feeding and stomatal morphology (feeding structure). The family is derived from bacteria feeders, and diverged to more than 30 genera, including several insect parasites, fungal feeders and predators^1,2^. Some diplogastrid genera have monomorphic short or long tube-like stomas without a conspicuous tooth, and feed on bacteria; others have various, short tube or barrel-like stomas with various-shaped teeth, ridges and denticles^1,2^. Interestingly, the general stomatal morphology is not clearly correlated with phylogeny, i.e., each form appears in separate lineages, suggesting genetic plasticity in stomatal morphology. In addition, many lineages of the family show stomatal dimorphism or polymorphism^3,4^. For example, the genus *Pristionchus* Kreis, a model system for developmental (phenotypic) plasticity, is a bacteria feeder with narrow short tube-like stoma (stenostomatous form), and its predatory form with wide barrel-shaped stoma (eurystomatous form) occurs according to environmental cues, e.g., shortage of bacteria and high population density^5^. The other diplogastrid genus, *Neodiplogaster* Cobb, is a group of fungal feeders, and its eurystomatous form is induced by environmental factors and the presence of potential prey species^4,6^.

*Pristionchus* and related genera have been investigated extensively in terms of the evolutionary biology of phenotypic plasticity and several other fields^5,7–10^. These omnivorous (dimorphic or polymorphic) species feed on bacteria or fungi and prey nematodes with two (or multiple) types of mouth forms.

In this study, during a field survey of insect-associated nematodes, a species of diplogastrid nematode was isolated from a dung beetle, *Onthophagus* sp. The nematode species showed omnivorous feeding, i.e., fungi, bacteria and prey nematodes, without clear stomatal dimorphism. The nematode is taxonomically described and illustrated as *Onthodiplogaster japonica* n. gen., n. sp. In addition, its feeding range was experimentally evaluated using cultured materials.

## Materials and methods

Several adult dung beetles, *Onthophagus* sp., were collected from a rotten mushroom, *Boletus violaceofuscus* W. F. Chiu (Agaricomycetes: Boletaceae) in the experimental *Quercus myrsinifolia* Blume stand in the Kansai Research Center, Forestry and Forest Products Research Institute, Kyoto, Japan (GPS: 34.94166666 N, 135.77361111 E, 77 m a.s.l.) in July 2021. The beetles were dissected individually on 2.0% water agar (2.0% agar without nutrients) and stored in the laboratory at room temperature (20–25 °C). The plates were observed for nematode propagation on the dissected beetle body for 2 weeks. Propagated nematodes were observed under a dissecting microscope (S8 Apo; Leica, Wetzlar, Germany) to determine their feeding habit, i.e., bacterial feeder, fungal feeder, or predator, and transferred to appropriate media using a stainless-steel insect pin (Insect Pin No. 00; Shiga Kontyu, Tokyo, Japan). The nematodes were considered bacteria feeders and were transferred to nematode growth medium (NGM) seeded with *Escherichia coli* strain OP50, a standard food for bacteriophagous nematodes. The successfully cultured nematode strain was stored as a laboratory culture with the code NKZ390.

### Morphological observation

Adult nematodes were recovered from 2-week-old cultures on *E. coli* OP50. Approximately one-third of the nematodes were used for morphological observation, according to Kanzaki^11^. There, about 100 males and females were casually observed to confirm the consistency of their morphology, and several individuals were examined closely. In addition, several individuals found feeding on fungi or preying on nematodes (*Acrobeloides* sp.) were examined for their stomatal morphology. Micrographs were obtained using a digital camera system (MC170 HD; Leica), and morphological drawings were made using a drawing tube connected to the microscope (Eclipse Ni; Nikon, Tokyo, Japan). Drawings and micrographs were used to construct figures with Photoshop Elements 2020 (Adobe, San Jose, CA, USA).

The remaining individuals were heat-killed at 55 °C for 30 s., and fixed in TAF (2.0% triethanolamine, 7.0% formalin) for 1 week. Nematodes were then processed to glycerine using the modified Seinhorst method^12^ and mounted in glycerine according to Maeseneer and d’Herde^13^. Mounted materials were used for morphometric analysis and deposited as type specimens.

### Scanning electron microscopy

The surface structures of the new species were observed by scanning electron microscopy (SEM). Adult females and males were collected from cultures on an NGM plate seeded with *E. coli* OP50 and transferred to M9 buffer. Nematodes were killed by heating on a hot plate (60 °C for 1 min.), and pre-fixed in 2% paraformaldehyde plus 2.5% glutaraldehyde in 0.1 M phosphate-buffered saline (PBS) for 15 h at 4 °C. Nematodes were post-fixed with 1% osmium tetroxide in 0.1 M PBS for 1.5 h and dehydrated in an ethanol series (25, 50, 70, 80, 90, and twice in 99.5% ethanol, 5 min each). Next, samples were immersed three times (5 min each) in 100% tert-butyl alcohol and lyophilised (JFD-310; JEOL, Tokyo, Japan). Samples were coated with osmium using a sputter coater (HPC-1SW; Vacuum Device, Ibaraki, Japan), and observed by SEM (JSM-6700F; JEOL).

### Feeding preference examination

The nematode was initially cultured on *E. coli* OP50. However, because of its typological similarity to the fungal feeding/predator *Neodiplogaster*, the feeding preference of *O. japonica* n. gen., n. sp. was examined using the prey species *Acrobeloides* sp., the standard food for *Seinura* spp. in our laboratory^14,15^, and two species of fungi, *Botrytis cinerea* Pers., standard food for *Neodiplogaster acaloleptae* Kanzaki^6,16^, and an unidentified Mucorales fungus isolated from a carrier *Onthophagus* sp.

Potential foods were inoculated on appropriate media, i.e., NGM was seeded with *E. coli* OP50 for *Acrobeloides* sp., and 2.0% malt extract agar (2.0% malt extract, 2.0% agar) was used for the two fungal species. Nematodes reared on *E. coli* OP50 were isolated by pouring deionised water on the media, rinsed three times with deionised water, and approximately 100 individuals of various stages were inoculated onto the potential foods. Therefore, *E. coli* OP50 was retained in the fungal culture. Three culture plates were used for each potential food. Thereafter, the feeding behaviour of the nematode was observed under a dissecting microscope and a light microscope, and its propagation was confirmed under a dissecting microscope.

### Molecular profiling and phylogeny

Several adult individuals were selected from cultured material (as described above), and transferred to nematode lysis solution^17,18^ individually for DNA extraction. These nematodes were digested at 55 °C for 20 min, and the lysates served as the templates for PCR. First, the materials were individually amplified and the D2-D3 expansion segments of the large subunit of ribosomal RNA (D2-D3 LSU) were sequenced according to Ye et al*.*^19^ to confirm the species identity. Thereafter, approximately 4 kb of ribosomal RNA including near-full-length small subunit (SSU; ~ 1.7 kb), internal transcribed spacer region (~ 0.9 kb) and D1-D4 expansion segments of the large subunit (D1-D4 LSU; ~ 1.4 kb) were determined following the methodology by Ekino et al*.*^20^ and Kanzaki et al*.*^21^. The partial sequence of mitochondrial cytochrome oxidase subunit I (mtCOI; 660 bp) was determined following the methodology of Kanzaki & Futai^22^. The sequences of the new species were deposited in the GenBank database under accession numbers LC721118 (rDNA) and LC721119 (mtCOI).

The SSU and D2-D3 LSU were subjected to molecular phylogenetic analysis. First, both sequences were compared with those in the database by BLAST homology searching (https://blast.ncbi.nlm.nih.gov/Blast.cgi?PROGRAM=blastn&PAGE_TYPE=BlastSearch&LINK_LOC=blasthome). According to the BLAST results and prior reports^23,24^, sequences for phylogenetic analysis were retrieved from the database (Table S1). Although the partial sequence of mtCOI gene was determined, the sequence was used only as a characteristic of the species, because the number of diplogastrid mtCOI sequences deposited in the GenBank database is not sufficient for phylogenetic analysis.

The sequences were aligned using MAFFT^25,26^ (http://align.bmr.kyushu-u.ac.jp/mafft/software/). The substitution model and parameters were determined by MEGA 7^27^ for each locus, and Bayesian phylogenetic analysis was conducted in MrBayes 3.2^28,29^; four chains were run for 4 × 10^6^ generations. Markov chains were sampled at intervals of 100 generations^30^. Two independent runs were performed, and, after confirming the convergence of runs and discarding the first 2 × 10^6^ generations as burn-in, the remaining topologies were used to generate a 50% majority-rule consensus tree.

## Results

### Recovered nematodes

In addition to *O. japonica* n. gen., n. sp. *Tokorhabditis* *atripennis *Ragsdale, Kanzaki, Yamashita & Shinya was isolated, and will be the subject of a future study.

### Phylogenetic status of *Onthodiplogaster japonica* n. gen., n. sp

**Figure 1:**
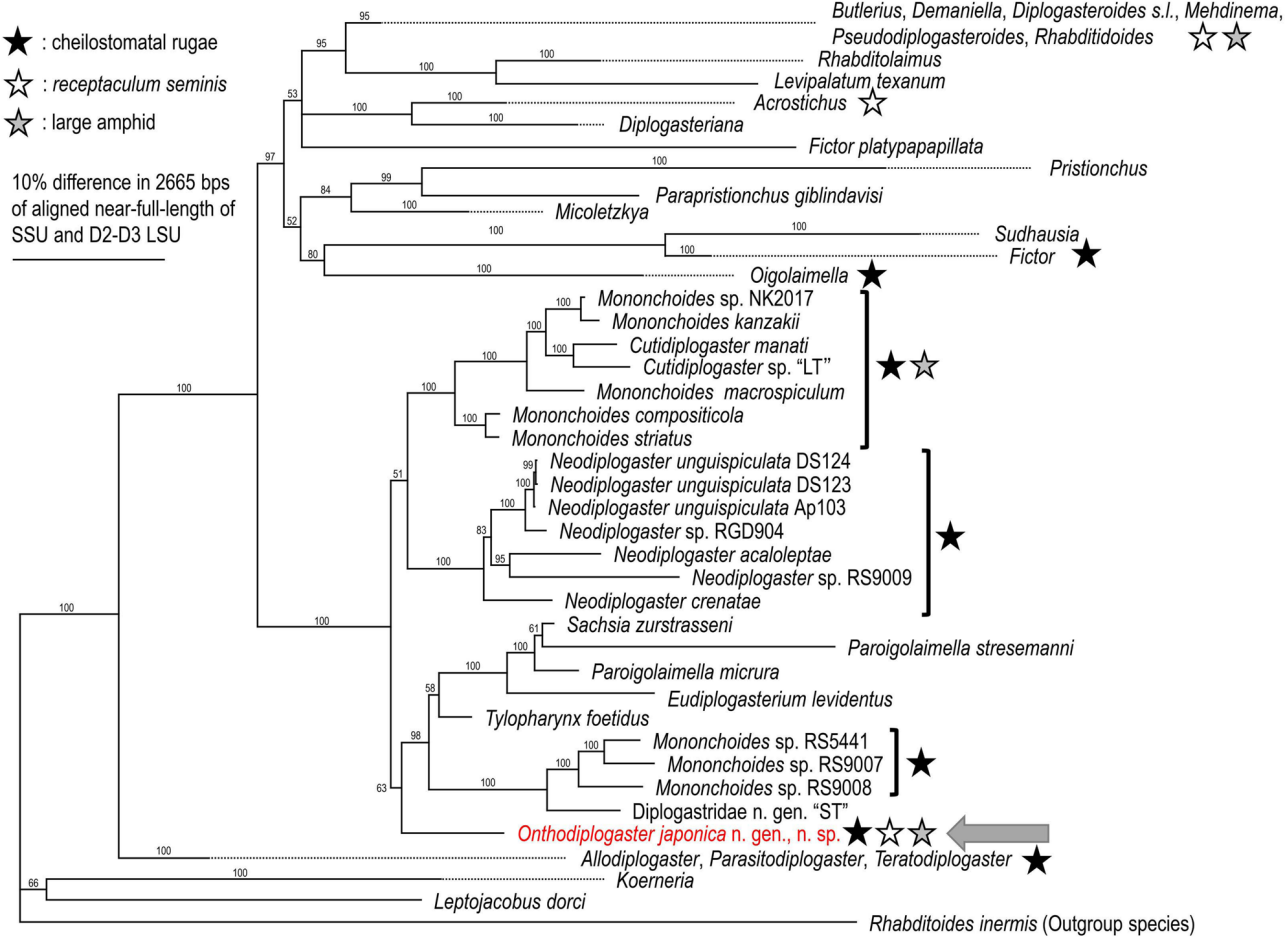
Molecular phylogenetic relationships among diplogastrid nematodes. The Bayesian tree inferred from near full length of SSU and D2-D3 LSU of ribosomal RNA genes. The GTR + G + I model was applied to both loci, and the parameters are as follows: AIC = 50,457.865; lnL = − 24,991.621; freqA = 0.25, freqC = 0.21, freqG = 0.27, freqT = 0.27; R(a) = 0.93, R(b) = 2.55, R(c) = 2.11, R(d) = 0.91, R(e) = 3.75, R(f) = 1.00; Pinva = 0.39; Shape = 0.58 for SSU, and AIC = 54,594.498; lnL = -27,059.552; freqA = 0.21, freqC = 0.22, freqG = 0.32, freqT = 0.25; R(a) = 0.46, R(b) = 1.69, R(c) = 0.90, R(d) = 0.43, R(e) = 3.42, R(f) = 1.00; Pinva = 0.21; Shape = 1.00 for D2–D3 LSU. Posterior probability (PP) values exceeding 50% are given on appropriate clades. Several clades far from the one new genus belong were compressed, and full phylogenetic tree is provided as Suppl. Fig. S1.

The phylogenetic status of *O. japonica* n. gen., n. sp. is shown in Fig. 1 and Suppl. Fig. S1. *Onthodiplogaster japonica* n. gen., n. sp. is included in the clade that harbours *Tylopharynx* de Man, *Mononchoides* Rahm, *Neodiplogaster*, *Sachsia* Meyl, *Paroigolaimella* Paramonov, *Eudiplogaserium* Meyl, *Cutidiplogaster* Fürst von Lieven, Uni, Ueda, Barbuto & Bain and the undescribed genus “ST” (short tail) Diplogastridae sp. isolated from manatee skin^24^ with maximal support (100% posterior probability). Several important typological characteristics, e.g., presence of *receptaculum seminis*, are indicated by asterisks in the phylogenetic tree (Fig. 1).

### Feeding preference of *Onthodiplogaster japonica* n. gen., n. sp

Nematodes showed active feeding and propagation on *Acrobeloides* sp. (Suppl. Movies S1, S2) and *B. cinerea* (Suppl. Movie S3). Although feeding behaviour was observed on the Mucorales fungus (Suppl. Movie S4), the nematode did not propagate on the fungus, and could not be subcultured. In addition, although *O. japonica* n. gen., n. sp. sometimes ate dead individuals, it was unclear whether the behaviour was cannibalism or scavenging. The behaviour of feeding on dead conspecific nematodes was not observed when co-cultured with *Acrobeloides* sp. on OP50.

The feeding behaviour, i.e., movements of stoma and pharynx and their coordination, was similar to that of *Neodiplogaster* and *Mononchoides* species^6,31^ regardless of food source.

### Taxonomic description of *Onthodiplogaster* n. gen

The generic epithet *Onthodiplogaster*, ‘*ontho*’ (= dung in Latin) + *Diplogaster* is derived from its carrier insect, *Onthophagus* spp., and its primary habitat. Although the type strain was isolated from a beetle on a rotten mushroom, the primary habitat of this phoretic nematode is usually determined by the carrier insect, and thus, the primary habitat of the nematode is hypothesized to be dung, carrion and other rotten materials which the dung beetle inhabits.

Diplogastridae. Body cylindrical, moderate to stout; typical for the family. Cuticle thick, with fine annulation and clear longitudinal striations. Lateral field weakly developed, sometimes difficult to distinguish from striations, but can be distinguished by lack of annulation, seemingly composed of two bands, i.e., three lines were observed. Six equal-sized lip sectors not clearly separated from each other, forming a dome shape, without clear constriction, continuous with body contour. Six short and papilliform labial sensilla present in male and female, and four small, papilliform cephalic papillae present in male, as typical for diplogastrid nematodes. Amphid large, ellipsoidal, located at the level of the posterior end of cheilostom. Stomatal dimorphism possibly present, i.e., the individuals feeding on other nematodes (predation) have somewhat wider stoma than microbe-feeding individuals, but no structural difference was observed. In addition, male has generally narrower stoma than female. Stoma separated into three sections: cheilo-, gymno- and stegostom. Cheilostom short, twice as wide as its depth, separated into 12 narrow plates (rugae). Gymnostom forming dorsally opened (notched) short cuticular tube with serrated anterior edge, separated into two (anterior and posterior) subsections, each derived from different arcade syncytia, but the margin is not clearly observed, and anterior end internally overlaps with the posterior end of cheilostom. Stegostom separated into three subsections: pro-meso-, meta- and telostegostom. Pro-mesostegostom not clearly cuticularised, internally overlapping with gymnostom. Metastegostom with a square- or diamond-shaped movable dorsal tooth, a square- or diamond-shaped right subventral tooth with several anterior spinous processes, and several left subventral ridges; anterior end of both teeth varies a little among individuals, from relatively simple triangular to claw-like shape; both teeth and ridges were connected to the stegostomatal cylinder (= telostegostom). Stegostom forming a cuticular cylinder, with a dorsal apodeme which appears as a narrow extension of dorsal telostegostomatal wall in lateral view, and wing or trapezoidal shape in ventral view. The posterior ends of right and left subventral sectors of telostegostom slightly expand, forming weakly developed apodeme. Dorsal pharyngeal gland orifice on the middle of dorsal tooth, i.e., the gland duct penetrates the dorsal tooth. Male gonad single, anteriorly reflexed. Male with nine pairs of papilliform genital papillae. Male tail with a spike of moderate length and sharply pointed tip. Female gonad paired, with oval or kidney-shaped *receptaculum seminis* at the dorsal side of the junction among vagina and anterior and posterior gonads. Female tail elongate conoid with pointed tip, i.e., without filiform tip.

- Phylum Nematoda Potts, 1932
- Class Chromadorea Inglis, 1983
- Order Rhabditida Chitwood, 1933

- Family Diplogastridae Micoletzky, 1922

*Onthodiplogaster* Kanzaki, Ikeda & Shinya, n. gen.

https://zoobank.org/NomenclaturalActs/60A942AC-1064-4F93-BAC1-71DA6341C30C.

Type species: *Onthodiplogaster japonica* n. gen., n. sp.

### Relationships

*Onthodiplogaster* n. gen. is characterised by its stomatal morphology, possessing 12 rugae in cheilostom and a cuticular stegostomatal cylinder, large ellipsoidal amphid and the presence of oval or kidney-shaped *receptaculum seminis* in the female.

These characteristics have been reported for other genera of the family. The cheilostomatal rugae are present in *Fictor* Paramonov, *Neodiplogaster*, *Mononchoides* and two species of *Allodiplogaster* Paramonov & Sobolev^1,32,33^. In addition, *Oigolaimella* Paramonov has cheilostom separated into many plates with a pointed anterior end referred to as a “corona”^34^. Large amphid is reported in *Demaniella* Steiner, *Goffartia* Hirschmann, *Sachsia* and several species of *Mononchoides*^1,35–37^. *Receptaculum seminis* is present in *Diplogasteroides* de Man sensu lato, *Pseudodiplogasteroides* Körner and *Acrostichus* Rahm^1,4^. Phylogenetically, *Onthodiplogaster* n. gen. is included in a clade with *Tylopharynx*, *Mononchoides*, *Neodiplogaster*, *Sachsia*, *Paroigolaimella*, *Eudiplogasterium*, and *Cutidiplogaster* (Fig. 1, Suppl. Fig. S1).

Although several genera have stomatal dimorphism (polymorphism), the diplogastrid genera are primarily separable from each other by their stomatal morphology^1^, and the new genus can be readily distinguished from *Tylopharynx*, *Sachsia*, *Paroigolaimella*, *Eudiplogasterium*, and *Cutidiplogaster* by the character.

*Onthodiplogaster* n. gen. is distinguished from *Tylopharynx* by its cheilostom, plated versus forming a ring (or short tube), gymnostom, forming ring or short tube with versus without anterior serratae, metastegostom, with square- to diamond-shaped dorsal and right subventral teeth and left subventral ridges versus triangular dorsal and right subventral teeth with claw-like tip and undeveloped left subventral sector, and stegostomatous apodeme weakly developed versus ball-shaped (rounded)^31^; from *Sachsia* by its cheilostom, plated versus forming a ring (or short tube), gymnostom, forming ring or short tube with versus without anterior serratae, metastegostom, with square- to diamond-shaped dorsal and right subventral teeth and left subventral ridges versus possessing only thorn-like dorsal tooth, and telostegostom forming deep cylinder versus short funnel shape^1,38^; from *Paroigolaimella* by cheilostom composed by 12 versus six plates, gymnostom forming ring (short tube) with anterior serratae versus simple tube, metastegostom with square- to diamond-shaped dorsal and right subventral teeth and left subventral ridges versus small triangular dorsal tooth and subventral warts (spiny plates), and telostegostom forming deep cylinder versus short funnel shape^1,4^; from *Eudiplogasterium* by its gymnostom forming ring (short tube) with anterior serratae versus simple tube without anterior serratae, metastegostom with square- to diamond-shaped dorsal and right subventral teeth and left subventral ridges versus small triangular dorsal tooth and small subventral ridges, and telostegostom forming deep cylinder versus short funnel shape^4^; and *Cutidiplogaster* by its cheilostom composed by 12 plates versus double-layered short tube, gymnostom forming ring with versus without anterior serratae, metastegostom with square- to diamond-shaped dorsal and right subventral teeth and left subventral ridges versus only small flap-like dorsal tooth, and telostegostom forming deep cylinder versus long annulated tube^39^.

Among these genera, *Onthodiplogaster* n. gen. is typologically close to *Mononchoides* and *Neodiplogaster*. These three genera share a characteristic stomatal composition, i.e., cheilostomatal rugae and telostegostomatal cuticular tube^1,2^. However, *Onthodiplogaster* n. gen. is distinguished from *Mononchoides* by the number of rugae, 12 versus various, but often more than 12, presence versus absence of *receptaculum seminis* and tail shape, absence versus presence of filiform tip^1,2,36,37,40^. The new genus is also distinguished from *Neodiplogaster* by its amphid shape, large ellipsoidal versus small pore-like, number of rugae, 12 versus 18, and presence versus absence of *receptaculum seminis*^1,2,6,41^.

### *Onthodiplogaster japonica* n. gen., n. sp

https://zoobank.org/NomenclaturalActs/D9F9DCEA-BCFE-4656-9A07-95B03B8C0191

Figures 2, 3, 4, 5, 6, 7, 8, 9 and 10; Supplementary Data 1–4.

**Figure 2:**
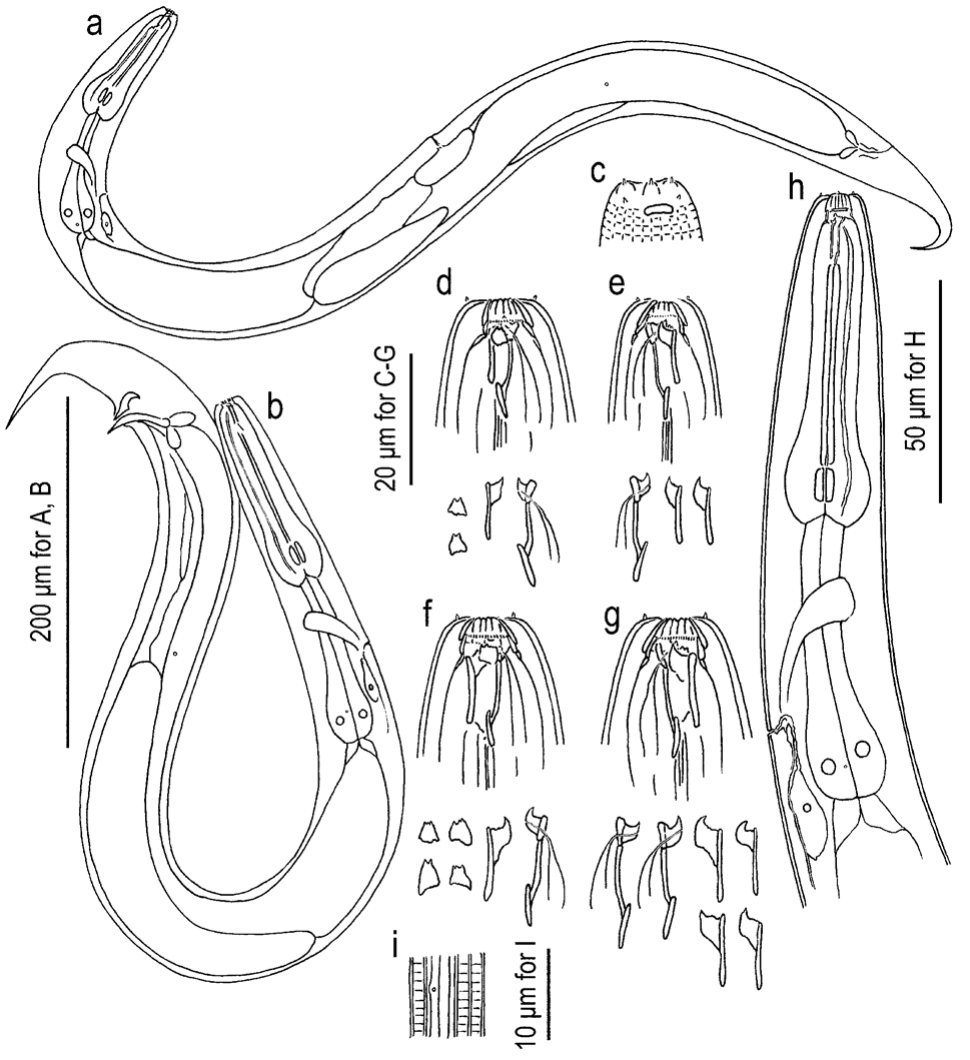
*Onthodiplogaster japonica* n. gen., n. sp. (**a**) Entire body of female in right lateral view; (**b**) entire body of male in right lateral view; (**c**) surface of lip region of male in left lateral view; (**d,e**) stomatal region of female feeding on *Botrytis cinerea* in left (**d**) and right (**e**) lateral views; (**f**,**g**) stomatal region of female feeding on *Acrobeloides* sp. in left (**f**) and right (**g**) lateral views; (**h**) pharyngeal region of female in left lateral view; (**i**) Body surface showing striation, lateral field and deirid in right lateral view. Left subventral ridge (with small triangular shape), right subventral and dorsal teeth (both form flag-like shape combined with telostegostomatal cylinder, and dorsal tooth with dorsal pharyngeal gland) are separately drawn below each subfigure (**d**–**g**).

**Figure 3:**
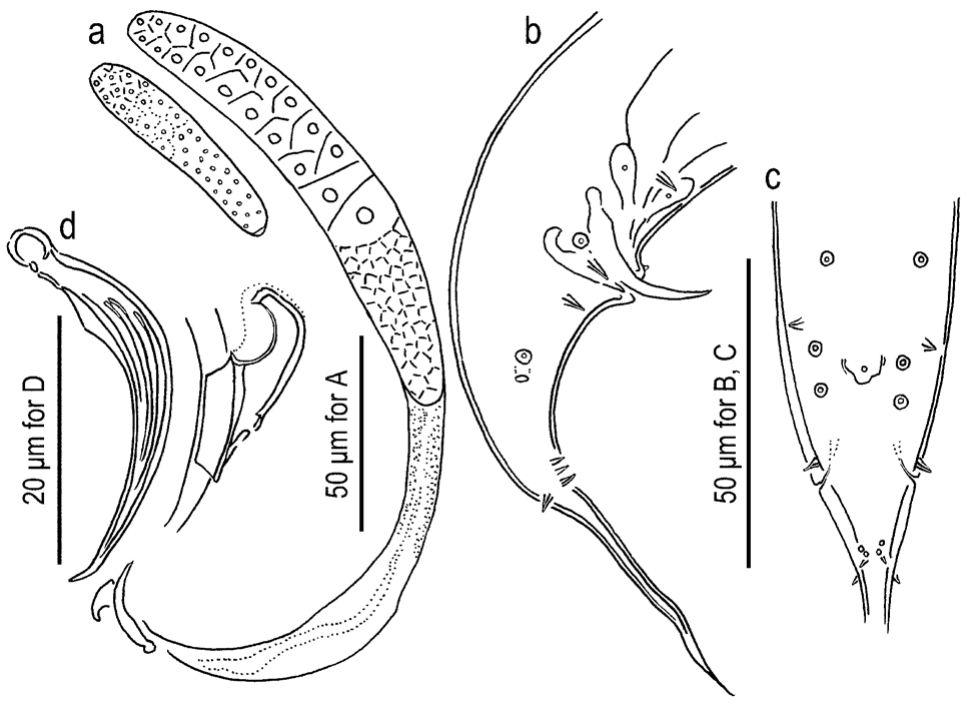
Adult male of *Onthodiplogaster japonica* n. gen., n. sp. (**a**) Gonad in left lateral view where reflexed part is drawn separately; (**b**) entire tail in right lateral view; (**c**) cloacal region in ventral view; (**d**) spicule and gubernaculum in left lateral view.

**Figure 4:**
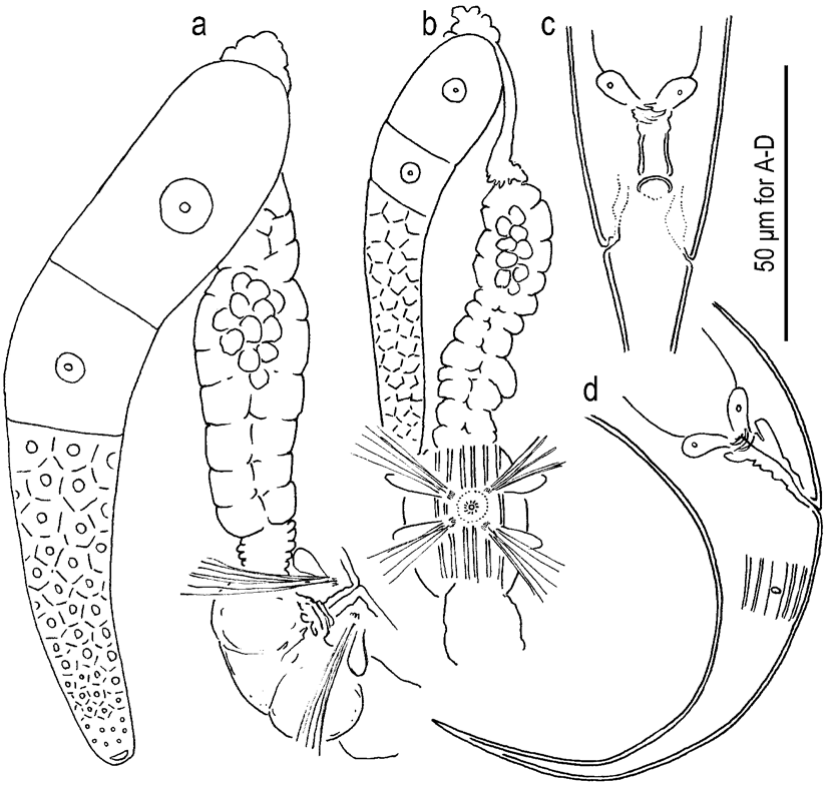
Adult female of *Onthodiplogaster japonica* n. gen., n. sp. (**a**) Anterior gonad in right lateral view; (**b**) anterior gonad in ventral view; (**c**) anal region in ventral view; (**d**) entire tail in right lateral view showing body surface structure in phasmid region.

**Figure 5:**
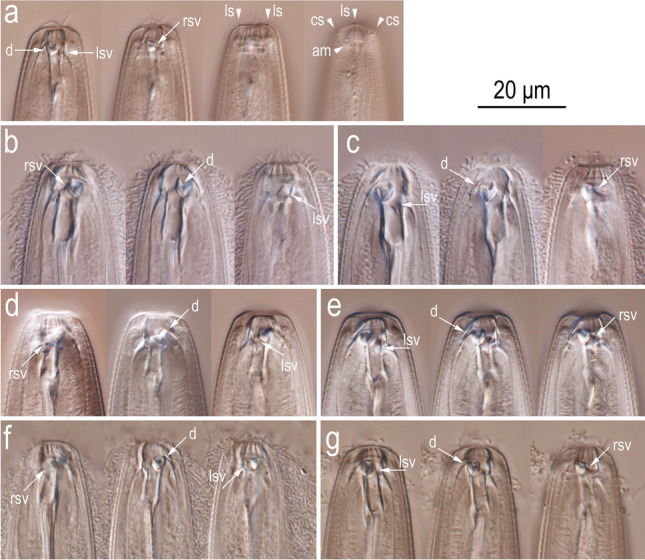
Light micrographs of stomatal region of *Onthodiplogaster japonica* n. gen., n. sp. (**a**) Right lateral view of male from fungal culture in four different focal planes; (**b,c**) left (**b**) and right (**c**) lateral views of predating female in three different focal planes; (**d,e**) left (**d**) and right (**e**) lateral views of bacteria feeding female in three different focal planes; (**f,g**) left (**f**) and right (**g**) lateral views of fungal feeding female in three different focal planes. Labels are as follows: am = amphid; cs = cephalic sensilla; d = dorsal tooth; lsv = left subventral ridge; ls = labial sensilla; rsv = right subventral tooth.

**Figure 6:**
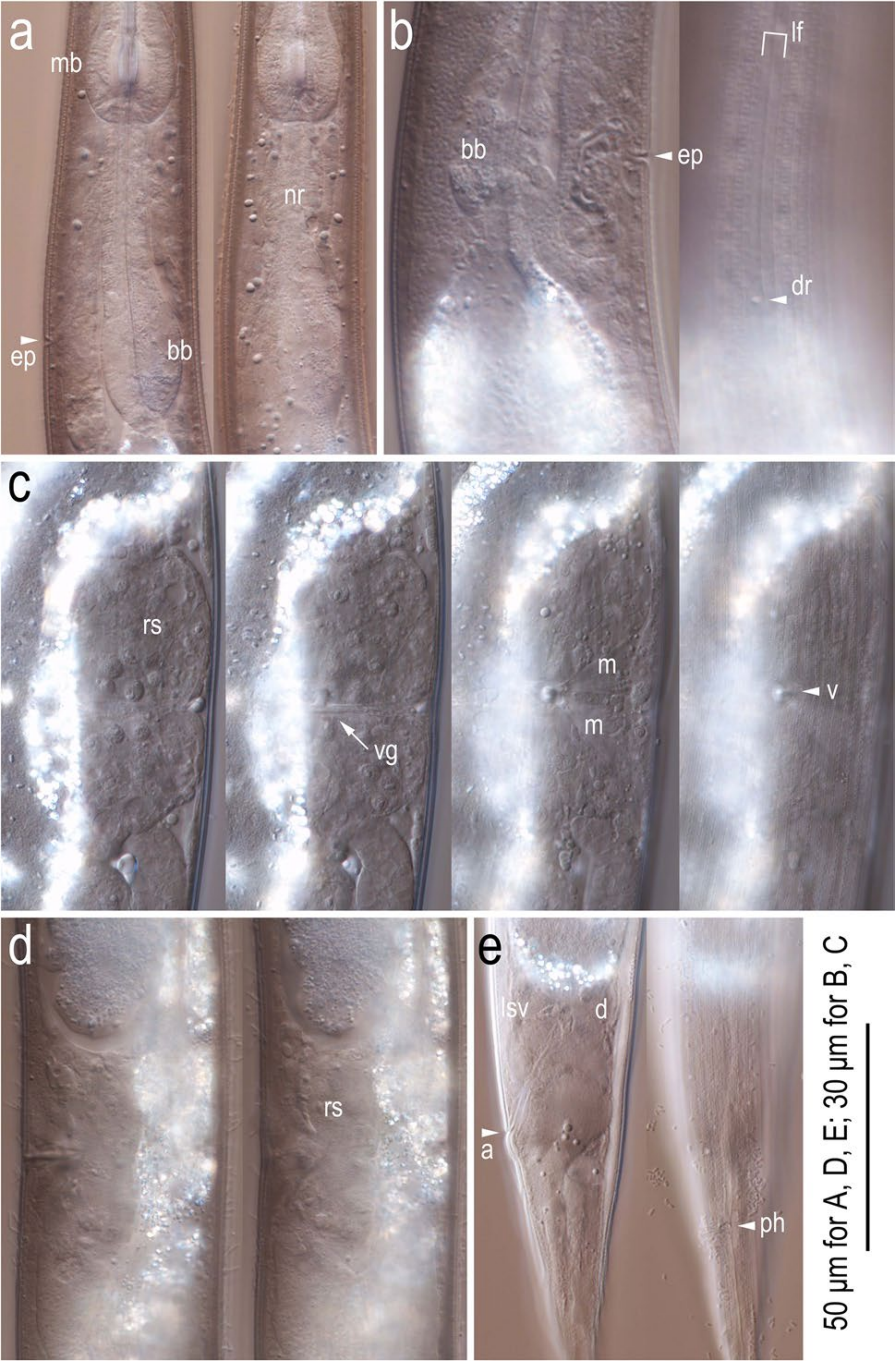
Light micrographs of anterior region and female characters of *Onthodiplogaster japonica* n. gen., n. sp. (**a**) Left lateral view of metacorpus to posterior pharynx region in two different focal planes; (**b**) right lateral view of basal bulb region in two different focal planes; (**c**) ventral view of vulval region in four different focal planes; (**d**) left lateral view of vulval region in two different focal planes; (**e**) left lateral view of anal region in two different focal planes. Labels are as follows: a = anus; bb = basal bulb; d = dorsal anal gland; dr = deirid; ep = secretory-excretory pore; lf = lateral field; lsv = left subventral anal gland; m = vulval muscle; mb = median bulb (metacorpus); ph = phasmid; rs = *receptaculum seminis*; v = vulva; vg = vagina.

**Figure 7:**
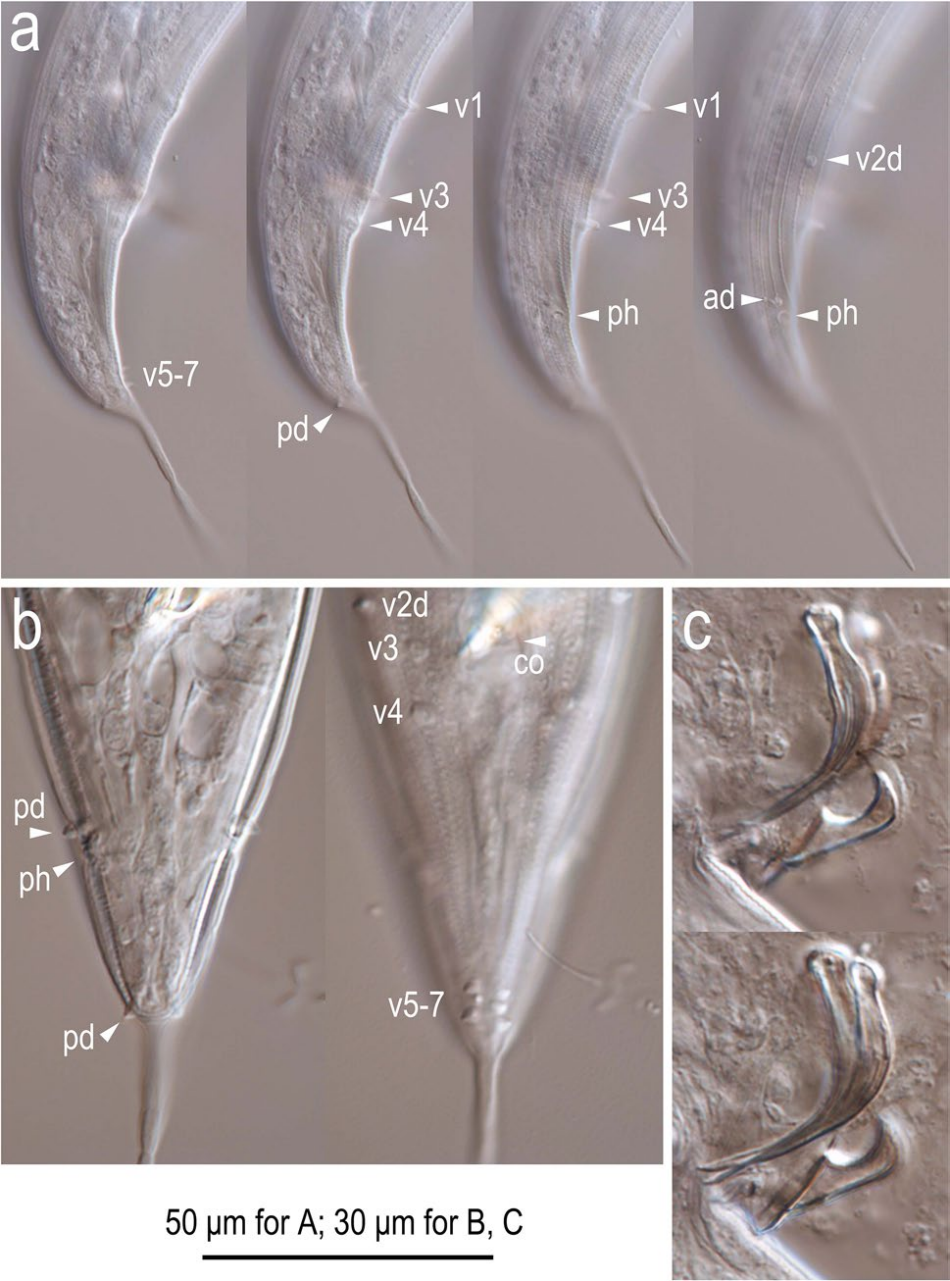
Light micrographs of male tail characters of *Onthodiplogaster japonica* n. gen., n. sp. (**a**) Right lateral view of entire tail in four different focal planes; (**b**) posterior region in two different focal planes; (**c**) flattened spicule and gubernaculum in two different focal planes. Labels are as follows: ph = phasmid; v + number, ad, pd = genital papillae according to the terminology by Sudhaus & Fürst von Lieven^1^.

**Figure 8:**
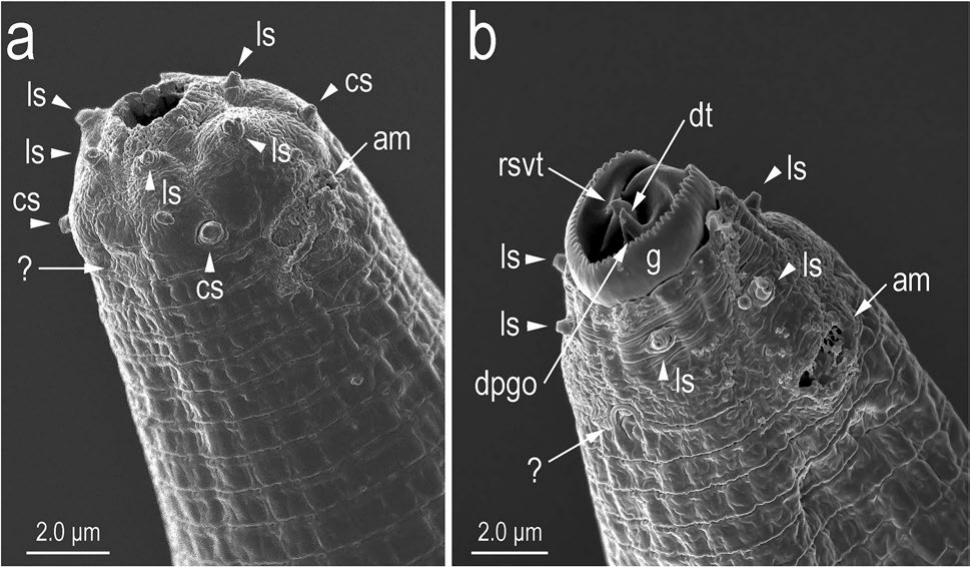
Scanning electron micrographs of stomatal region in left subventral view. (**a**) Male; (**b**) female with everted gymnostom and stegostom. Labels are as follows: am = amphid; cs = cephalic sensilla; dpgo = dorsal pharyngeal gland orifice; dt = dorsal tooth; g = gymnostom; ls = labial sensilla; rsvt = right subventral tooth; ? = an indentation with unknown function.

**Figure 9:**
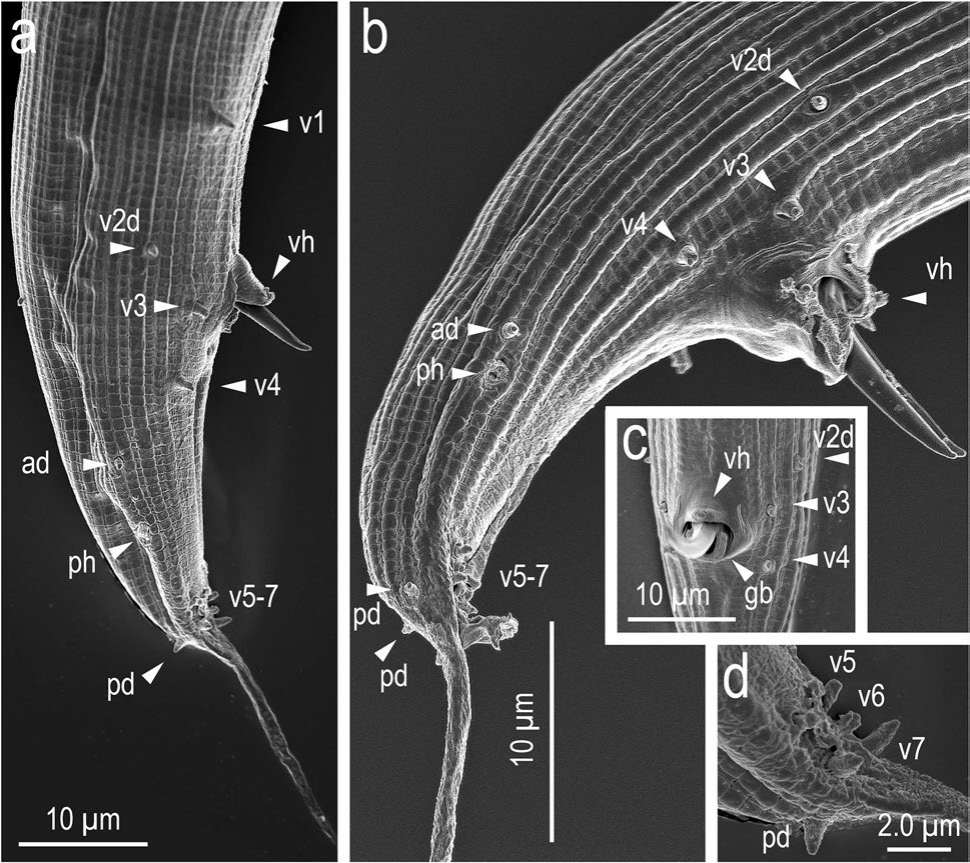
Scanning electron micrographs of male tail characters. (**a,b**) Entire tail in right lateral view of two different individuals; (**c**) ventral view of cloacal region; (**d**) right subventral view of posterior region. Labels are as follows: ph = phasmid; v + number, ad, pd = genital papillae according to the terminology by Sudhaus & Fürst von Lieven^1^; vh = ventral hook (ventral single papilla). Tail tip is out of the range of the micrograph in Fig. 9a.

**Figure 10:**
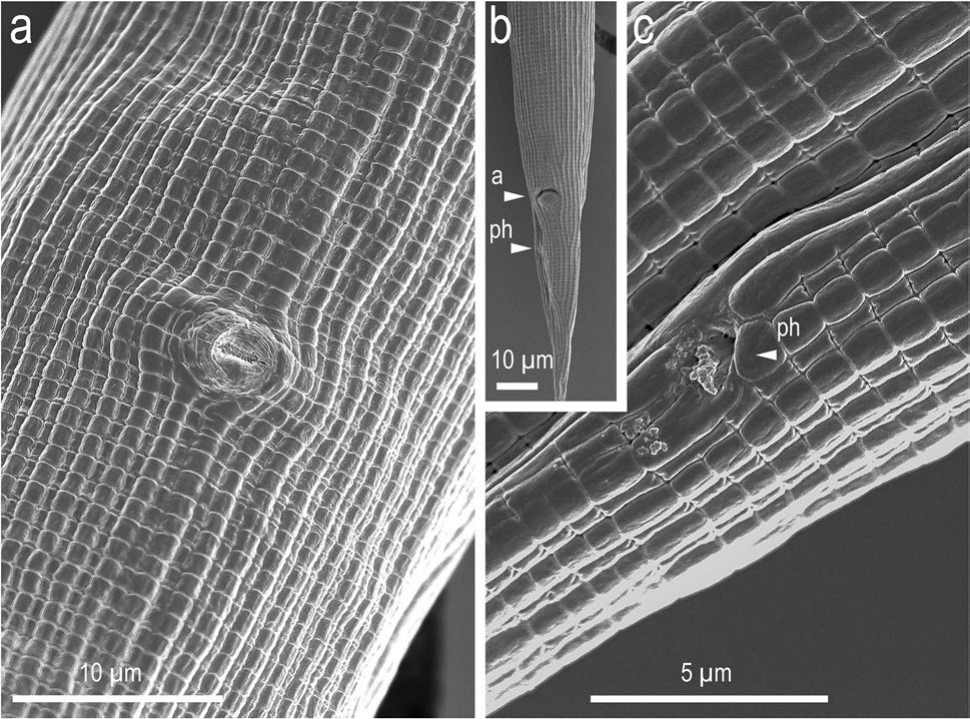
Scanning electron micrographs of female characters. (**a**) Ventral view of vulva; (**b**) ventral view of entire tail; (**c**) right lateral view of phasmid region. Labels are as follows: a = anus; ph = phasmid.

Species epithet is derived from its type locality, Japan.

### Measurements

See Table 1.

**Table 1:** Morphometrics of *Onthodiplogaster japonica* n. gen., n sp. All measurements are in μm and in the form: mean ± s.d. (range).

Character	Male		Female
	Holotype	Paratypes	Paratypes
n	–	9	10
L	532	519 ± 38 (450–589)	652 ± 62 (528–723)
a	18.9	19.7 ± 1.9 (16.4–22.4)	18.8 ± 1.8 (16.3–21.9)
b	4.8	4.8 ± 0.3 (4.2–5.1)	5.2 ± 0.4 (4.4–5.6)
C^a^	7.0	7.0 ± 0.5 (6.0–7.7)	5.7 ± 0.2 (5.4–6.1)
c′^a^	4.0	3.7 ± 0.4 (3.2–4.4)	5.5 ± 0.4 (4.8–6.1)
T^b^ or V	53.2	55.6 ± 5.1 (46.5–64.4)	50.6 ± 0.7 (49.7–51.7)
Maximum body diam.^c^	28	27 ± 2.9 (22–31)	35 ± 5.9 (24–42)
Stoma opening diam	2.2	2.5 ± 0.3 (2.1–3.2)	2.9 ± 0.4 (2.3–3.6)
Metastegostomatal (maximum) diam	4.3	4.2 ± 0.2 (3.9–4.6)	5.3 ± 0.3 (4.8–5.7)
Telostegostomatal diam.	2.5	2.3 ± 0.2 (1.8–2.5)	3.0 ± 0.2 (2.5–3.2)
Stoma depth	12.3	12.0 ± 0.3 (11.4–12.5)	13.9 ± 0.9 (12.5–15.4)
Stoma depth/maximum diam. ratio	2.9	2.9 ± 0.2 (2.6–3.1)	2.7 ± 0.2 (2.4–3.0)
Anterior pharynx length	49	48 ± 2.2 (44–51)	55 ± 1.6 (52–58)
Posterior pharynx length	49	49 ± 3.7 (41–53)	56 ± 3.4 (51–61)
Anterior/posterior pharynx length ratio	1.0	1.0 ± 0.1 (0.9–1.1)	1.0 ± 0.1 (0.9–1.1)
Median bulb diam.	13.9	14.0 ± 0.6 (13.2–15.0)	17.3 ± 1.3 (15.0–18.6)
Basal bulb diam.	11.4	11.0 ± 0.7 (10.0–12.1)	14.3 ± 1.2 (12.5–16.4)
Nerve ring from anterior end	77	75 ± 3.3 (69–81)	84 ± 3.0 (80–91)
Secretory-excretory pore from anterior end	102	98 ± 6.2 (89–108)	107 ± 6.1 (100–116)
Cloacal or anal body diam.	19.1	19.9 ± 1.0 (18.4–21.3)	20.6 ± 1.0 (18.4–22.0)
Tail length^a^	76	74 ± 8.0 (64–89)	114 ± 8.9 (98–126)
Tail spike length	38	38 ± 5.9 (30–48)	–
% of tail spike to whole tail	50.5	50.8 ± 3.2 (46.4–54.9)	–
Relative length of tail spike to cloacal body diam.	2.0	1.9 ± 0.3 (1.5–2.5)	
Whole gonad length	283	289 ± 36 (223–350)	–
Reflex part of testis	47	45 ± 5.4 (34–52)	–
*Vas deferens* length^b^	86	84 ± 8.1 (77–99)	–
% of *vas deferens* to whole gonad	30.6	29.5 ± 4.1 (22.2–35.6)	–
Spicule length (chord)	29	27 ± 2.0 (25–31)	–
Spicule length (curve)	33	32 ± 2.3 (27–35)	–
Gubernaculum length (chord)	14.4	15.4 ± 1.1 (14.2–17.7)	–
Gubernaculum width	6.2	6.1 ± 0.3 (5.7–6.4)	–
Anterior ovary length	–	–	122 ± 27 (69–160)
Posterior ovary length	–	–	115 ± 23 (68–153)
Anterior/posterior ovary length ratio	–	–	1.07 ± 0.16 (1.00–1.38)
*Receptaculum seminis* length	–	–	41 ± 5.1 (32–50)
*Receptaculum seminis*/vulval body diam. ratio	–	–	1.17 ± 0.11 (1.00–1.38)
Phasmid from anus	–	–	14.4 ± 2.2 (11.3–17.7)
Relative position of phasmid to anal body diam.^d^	–	–	0.70 ± 0.11 (0.53–.083)
Relative position of phasmid to tail length^e^	–	–	12.6 ± 1.8 (9.6–15.3)

^a^Tail length including tail spike.

^b^Gonad length including reflex part and *vas deferens.*

^c^Body diam. is maximum at vulval part in hermaphrodite and female (vulval body diam. = maximum body diam).

^d^Calculated as anus-phasmid distance/anal body diam.

^e^Calculated as 100 × anus-phasmid distance/whole tail length.

### Description

To avoid redundancy with previous diplogastrid species descriptions, a short description on the diagnostic characters is presented here, and a detailed description in traditional telegraph style is provided as Supplementary text S1. In addition, stomatal elements mentioned in the generic and species descriptions and Figs. 2 and 5 are illustrated in Suppl. Fig. S2.

#### Adults

Medium-to-small sized species as the family, body length 450–589 μm in males and 528–723 μm in females. Body shape, body surface structure and stomatal structure as described for generic characteristics, but a circular or horseshoe-shaped indentation is observed on ventral side at the level of amphid. Pharynx composed by muscular anterior part with cuticular inner lining and glandular posterior part. Nerve ring at the level of mid-isthmus. Secretory–excretory pore at level of basal bulb to cardia. Deirids slightly posterior to secretory–excretory pore. Lateral glands not observed.

#### Males

Body ventrally arcuate, strongly ventrally curved at tail region when killed by heat. Testis single, on the right ventral of intestine, with typical structure of the family. Postdeirid around the anterior end of *vas deferens*. Spicules separate, smoothly curved in ventral view; smoothly ventrally arcuate in lateral view, with rounded to roundish squared manubrium; lamina/calomus complex ventrally expanded at one-fifth to one-fourth length from anterior end. Gubernaculum, about half of spicule in length, ear-like shape in lateral view; anterior part forming ventrally curved extension with blunt tip; posterior half dorsally enveloping spicules. Dorsal side of gubernaculum well sclerotised. Tail conical, with a sharply pointed spike which is approximately 1.5–2.5 cloacal body diameter (CBD) in length. In total, 19 papilliform genital papillae, i.e., one small, ventral, single papilla on anterior cloacal lip, and nine pairs, present. Nine pairs of genital papillae and a pair of phasmids present, and arranged as < v1, v2d, v3 / v4, ad, v5–v7, pd > in the terminology of Sudhaus & Fürst von Lieven^1^, where subventral v1 approximately 1 CBD anterior to cloacal opening (CO); laterally located v2d slightly anterior to CO; subventral P3 almost adcloacal; subventral P4 less than one-third CBD posterior to CO, i.e. v2d, v3, CO and v4 are close to each other; cloacal slit and P4 are close to each other; laterally located at approximately 1 CBD posterior to CO; v5–v7 forming triplet, and the central one (v6) slightly more ventrally located than the other two; subdorsally directed pd located at level of or slightly posterior to v7. Anterior five pairs (v1–ad) almost equal in size, rather large and conspicuous; v5 and v6 very small; and v7 and pd small but larger than v5 and v6. Phasmid conspicuous, forming ellipsoidal pore, located slightly posterior to ad.

#### Females

Slightly and smoothly arcuate ventrally when killed by heat. Gonad didelphic, amphidelphic with typical structure of the family, but a *receptaculum seminis* forms an independent branch. Vagina pore-like in ventral view, without flap apparatus. *Receptaculum seminis* oval or kidney-shaped in lateral view, rounded in ventral view; overlapping the dorsal side of uterus; sometimes harbours tightly packed sperm (spermatophore). Postdeirid at the level of the reflection of posterior gonad. Phasmid located less than one anal body diameter posterior to anus. Tail smoothly tapering or slightly elongate conical, with pointed terminus.

#### Type host and locality

New species was isolated from *Onthophagus* sp., collected from a rotten mushroom, *Boletus violaceofuscus*, in the experimental stand of the Kansai Research Center, Forestry and Forest Products Research Institute, Kyoto, Japan (GPS: 34.94166666 N, 135.77361111 E, 77 m a.s.l.) in July 2021.

#### Type material

Holotype male (slide number: T-775t) four paratype males (T-7718p and T-7721p) and five paratype females (T-7722p and T-7726p) deposited in the USDA Nematode Collection, Beltsville, MD, USA; five paratype males (*Onthodiplogaster japonica* M01–05) and five paratype females (*Onthodiplogaster japonica* F01–05), deposited in the Forest Pathology Laboratory Collection at Forestry and Forest Products Research Institute, Tsukuba, Japan.

## Discussion

*Onthodiplogaster* n. gen. is described based on its morphological characteristics: (1) presence of oval or kidney-shaped *receptaculum seminis*, (2) stoma with 12-plated cheilostomatal rugae, gymnostom with serrated and notched anterior edge and stegostomatal cuticular cylinder, and (3) large and ellipsoidal amphid. The new genus is described based on the combination of the characters, i.e., without a clear genus-specific apomorphy. Therefore, in some cases, the justification of the generic status could be typologically challenging, e.g., the species could be placed into the typologically closest genus, *Mononchoides*. However, considering its phylogenetic status, i.e., clearly separable from this typologically similar genus, and the paraphyly of the genus *Mononchoides* (Fig. 1, Suppl. Fig. S1), we consider that the new species, *O. japonica* n. gen., n. sp. is suitable for classification as a separate genus to avoid confusion in future systematic studies.

Interestingly, each of these morphological characteristics, has been reported in genera phylogenetically separate from the clade , although the gymnostomatal shape (dorsal notch) has not been confirmed in other genera. This suggests that these characteristics can occur independently in different clades of diplogastrids.

The oval or kidney-shaped *receptaculum seminis* is a generic diagnostic characteristic of *Acrostichus*^1,2,42^, and is confirmed in several *Diplogasteroides s. l.* species previously considered an independent genus, *Pseudodiplogaster*^43,44^. Also, although the shape is different, a long and slender *receptaculum seminis* is present in several *Diplogasteroides* (previous *Rhabdontolaimus* Fuchs) and *Pseudodiplogasteroides*^45,46^. Considering their phylogenetic relationships, i.e., *Onthodiplogaster* n. gen., *Acrostichus*, *Diplogasteroides* (*Pseudodiplogaster*), *Diplogasteroides* (*Rhabdontolaimus*), and *Pseudodiplogasteroides* are phylogenetically separated (not monophyletic)^4^ (Fig. 1, Suppl. Fig. S1), these characteristics occurred independently at least five times. In all cases, the structure harbours tightly packed sperm (spermatophore)^44,47,48^. Therefore, the structure, as represented in its morphological terminology, receives the spermatophore during copulation, and stores it until the sperm migrate to spermatheca. However, the adaptive significance of the structure is unknown. For example, close relatives (= phylogenetically neighbouring genera) do not have this structure^1,4^.

The composition of the stoma of the new genus is shared with its relatively close relatives, *Mononchoides* and *Neodiplogaster*, i.e., narrow cheilostomatal plates (rugae), teeth and ridges in metastegostom and telostegostomatal cylinder. Because the new genus is relatively basal to the clade, and members of the clade have highly variable stomatal forms—e.g., a cheilostom ring in *Sachsia*, *Tylopharynx* and *Cutidiplogaster*^1,38,39,49^, and plates in *Paroigolaimella*^1,50^—the stomatal shape shared by *Onthodiplogaster* n. gen., *Mononchoides* and *Neodiplogaster* could be a plesiomorphic character of the clade containing these genera. Further developmental and ecological studies are necessary to understand the evolution of stomatal morphology.

A large amphid is shared by the new genus, some *Mononchoides* species, *Sachsia*, *Demaniella* and *Goffartia*; *Demaniella* belongs to a different clade than the new genus (Fig. 1, Suppl. Fig. S1), and the molecular phylogenetic status of *Goffartia* is unknown. In addition, several *Allodiplogaster* species associated with dung beetles, e.g., *A. henrichae* (Sachs) Paramonov & Sobolev, and several freshwater species, e.g., *A. carinata* (Zullini) Kanzaki, Ragsdale & Giblin-Davis, have relatively large amphids^45,51^. An amphid is an opening of the chemosensory organ that enables perception of water-soluble substances^52–54^; therefore, the large opening of the amphid suggests that the nematode is highly sensitive to chemical (environmental) cues under humid conditions. The genera and species with large amphids are commonly found in nutrient-rich or humid environments, i.e., rotten plant materials (compost and humus), dung, carrion, and polluted water^1,36,45,55^. Therefore, the large amphid is hypothesised to be an adaptation to heterologous environments with high nutrient loads and humidity. This is because the substrates, e.g., dung and carrion, encompass heterologous conditions, contain many microbes and nematodes and are decomposed quickly^56,57^. Accordingly, these nematodes need to recognise environmental changes and respond to them by effectively receiving chemical signals to distinguish nutrients (foods) and natural enemies using their large amphids. However, several other nematode groups living in similar conditions do not have large amphids, and so may have different ways of adapting to the environment. *Onthodiplogaster japonica* n. gen., n. sp. showed little conspecific predation (cannibalism) in co-culture with *Acrobeloides* sp. The diplogastrid nematode *Pristionchus pacificus* Sommer, Carta, Kim & Sternberg shows stomatal dimorphism and avoids cannibalism by chemically recognising the species^9,58^. Similarly, the large amphid of *O. japonica* n. gen., n. sp. may enable recognition of conspecific individuals for mating and avoiding cannibalism.

A circular or horseshoe-shaped indentation was observed at the amphid level (Fig. 8). This structure was clearer in females and could not be confirmed by light microscopy. Also, the presence or absence of the structure on the dorsal side was not confirmed. Because such a structure has not been reported in other diplogastrid nematodes, its function is unknown, and further examinations of the structure, including whether it is present or an artefact, are necessary.

An interesting biological characteristic of *O. japonica* n. gen., n. sp. is omnivory, i.e., the species feeds on fungi, bacteria and other nematodes. Omnivory has also been reported in other diplogastrid species, including the bacteriophagous stenostomatous and predatory eurystomatous forms of *Pristionchus* spp.^5^, and fungal feeding stenostomatous and predatory eurystomatous forms of *Neodiplogaster* spp.^6^. In addition, an aphelenchoidid fungal feeder, *Bursaphelenchus sinensis* Marinari Palmisano, Ambrogioni, Tomiczek & Brandstetter, and its close relatives show stylet dimorphism to produce a predatory form^59–62^. However, *O. japonica* n. gen., n. sp. is the only omnivore that feeds on three food sources (fungi, bacteria and nematodes) with a nearly (or totally) monomorphic stoma. If the species is truly monomorphic, that could be an adaptation to heterologous and short-lived substrates, i.e., the species can feed on various foods without stomatal dimorphism, which requires at least one generation to change the stomatal morphology. A similar pattern of omnivory has been reported in *Mononchoides kanzakii* Mahboob, Bashir, Asif, Nazir, Jahan & Tahseen and its close relative *M. composticola* Steel, Moens, Scholaert, Boshoff, Houthoofd & Bert. These bacteriophagous/predatory nematodes are found in nutrient-rich environments, e.g., humus and manure, in India, and sometimes associated with dung beetles^63^. Their feeding pattern could be a similar adaptation to habitat as in *O. japonica* n. gen., n. sp.

Interestingly, *O. japonica* n. gen., n. sp. fed on, but the feeding was not active, and did not propagate on, Mucorales fungus. The hyphae of Mucorales fungus may contain repellent and/or toxic substance(s) for the nematode. Mucorales fungi generally grow rapidly and consume nutrients. Therefore, the nematode’s feeding on the fungus could damage hyphae and thereby constrain fungal growth.

Further investigation of the stomatal structural morphology, development and genetics of *O. japonica* n. gen., n. sp. will provide insight into its adaptation and phenotypic plasticity for comparison with *Pristionchus*, a model for developmental plasticity.

## Supplementary Information

Supplementary Video 1.

Supplementary Video 2.

Supplementary Video 3.

Supplementary Video 4.

Supplementary Information 1.

## Data Availability

The molecular sequences generated during the current study are available in the GenBank database with accession numbers, LC721118 and LC721119. The accession numbers of the other sequences phylogenetically analyzed in the current study are summarized in Supplementary Table S1. This publication and its nomenclatural acts have been registered in Zoobank (www. zoobank.org), the official registry of the International Commission on Zoological Nomenclature. The LSID (Life Science Identifier) of the article is http://zoobank.org/References/01E97499-085D-454C-A10F-6385D7E3BF56.
