# Supplementary material for: Onthodiplogaster japonica n. gen., n. sp. (Rhabditida: Diplogastridae) isolated from Onthophagus sp. (Coleoptera: Scarabaeidae) from Japan
Source: Sci Rep. 2023 Apr 20;13:6470. doi: 10.1038/s41598-023-33586-1 (PMC10119125; doi:10.1038/s41598-023-33586-1)
Supplement: Supplementary file 5 — Supplementary Information 1. [file 41598_2023_33586_MOESM5_ESM.pdf]

Full typological description for *Onthodiplogaster japonica* n. gen., n. sp.

Description in a traditional telegraphic style was not presented in main text to avoid overlapping of fixed phrases with previous species descriptions of typologically similar diplogastrid species. Therefore, the full description is given herein as supplementary material.

***Onthodiplogaster japonica** *n. gen., n. sp.**

Figures 2–10; Supplementary Data 1–4.

Species epithet is derived from its type locality, Japan.

Measurements

See Table 1.

Description

Adults. Medium-to-small sized species as the family, 450–589 μm in males and 528–723 μm in females. Body shape, body surface structure and stomatal structure as described for generic characteristics, but a circular or horseshoe-shaped indentation is observed on ventral side at the level of amphid. Anterior part of pharynx (= pro- and metacorpus) and posterior pharynx (isthmus and basal bulb) of almost identical length. Procorpus very muscular, with conspicuous internal lining, occupying about half of corresponding body diameter. Metacorpus very muscular, forming well-developed median bulb. Isthmus narrow, not muscular. Basal bulb glandular. Pharyngo-intestinal junction clearly observed, well developed. Nerve ring usually surrounding middle part of isthmus. Secretory–excretory pore visible, but not conspicuous, ventrally located at level of basal bulb to pharyngo-intestinal junction. Deirids observed laterally, at the level of metacorpus to slightly posterior to pharyngo-intestinal junction, *i.e.*, slightly posterior to secretory–excretory pore. Postdeirid present, on latero-dorsal side of the body; the position is described for males and females separately. Lateral glands not observed.

Males. Body ventrally arcuate, strongly ventrally curved at tail region when killed by heat. Testis single, on the right ventral of intestine, anterior part reflexed to right side. Spermatogonia arranged in multiple (three to five) rows in reflexed part, well-developed spermatocytes arranged as multiple (one to three) rows in anterior two-thirds of main branch, and mature amoeboid spermatids arranged in multiple rows in remaining, proximal part of gonad. *Vas deferens* occupying about one-third of total gonad length. Posterior end of gonad (= posterior end of *vas deferens*) and intestine fused to form a cloacal tube. Postdeirid around the anterior end of *vas deferens*. Spicules paired, separate. Spicules smoothly curved in ventral view, adjacent to each other for distal third of their

length, each smoothly tapering to bluntly pointed distal end. Spicule in lateral view smoothly ventrally arcuate, giving spicule about 90° curvature, rounded to roundish squared manubrium present at anterior end; lamina/calomus complex ventrally expanded at one-fifth to one-fourth length from anterior end, then smoothly tapering to bluntly pointed distal end. Gubernaculum conspicuous, about half of spicule in length, ear-like shape in lateral view; anterior part forming ventrally curved extension with blunt tip; posterior half dorsally enveloping spicules. Dorsal side of gubernaculum well sclerotised. Distal end of gubernaculum possessing short blunt process on dorsal side in lateral view. Tail conical, with a sharply pointed spike which is approximately 1.5 cloacal body diameter (CBD) in length, occupying about half of tail length. In total, 19 papilliform genital papillae, *i.e.*, one small, ventral, single papilla on anterior cloacal lip, and nine pairs, present. Nine pairs of genital papillae and a pair of phasmids present, and arranged as <v1, v2d, v3 / v4, ad, v5–v7, pd> in the terminology of Sudhaus & Fürst von Lieven [1], where subventral v1 approximately 1 CBD anterior to cloacal opening (CO); laterally located v2d slightly anterior to CO; subventral P3 almost adcloacal; subventral P4 less than one-third CBD posterior to CO, *i.e.* v2d, v3, CO and v4 are close to each other; cloacal slit and P4 are close to each other; laterally located at approximately 1 CBD posterior to CO; v5–v7 forming triplet, and the central one (v6) slightly more ventrally located than the other two; subdorsally directed pd located at level of or slightly posterior to v7. Anterior five pairs (v1–ad) almost equal in size, rather large and conspicuous; v5 and v6 very small; and v7 and pd small but larger than v5 and v6, *i.e.*, intermediate between anterior pairs and v5/v6. Phasmid conspicuous, forming ellipsoidal pore, located slightly posterior to ad. Bursa or bursal flap absent.

Females. Slightly and smoothly arcuate ventrally when killed by heat. Gonad didelphic, amphidelphic. Each genital system arranged from vulva/vagina as *receptaculum seminis*, uterus, spermatheca, oviduct, connection tissue and ovary; where uterus to ovary form a single tube, and *receptaculum seminis* forms an independent branch. Because anterior and posterior gonads are structurally identical and symmetrical to each other, *i.e.*, anterior and posterior gonads extend on the right and left of intestine, only anterior gonad is described here from vagina/vulva to ovary. Vagina pore-like in ventral view, without flap apparatus. Vagina perpendicular to body wall, surrounded by sclerotised tissue. Vulval muscle forming X-shape in ventral view, relatively conspicuous. Four vaginal glands visible overlapping with vulval muscle. *Receptaculum seminis* oval or kidney-shaped in lateral view, rounded in ventral view; overlapping the dorsal side of uterus; sometimes harbours tightly packed sperm (spermatophore). Uterus to connection tissue extending ventrally and anteriorly on right of intestine and with a totally reflexed (= antidromous reflexion)

ovary extending dorsally. Uterus thick-walled, but not clearly observed because it is often masked by *receptaculum seminis*. Middle part of main gonad branch functions as spermatheca where well-developed sperm often present; composed by large rounded-flattened cells. Oviduct simple tube between spermatheca and connection tissue. Connection tissue consisting of oval-shaped cells, somewhat crustaformeria-like, connecting ovary and oviduct. Oocytes mostly arranged in multiple (two to five) rows in distal half, and well-developed oocytes arranged in single row, where the most developed (anterior) oocyte appears darker and more glandular than the other oocytes. Postdeirid at the level of the reflection of posterior gonad. Rectum approximately 1 anal body diameter (ABD) long, intestinal-rectal junction surrounded by well-developed sphincter muscle. Three rectal glands, two ventral and one dorsal, present. Anus in form of dome-shaped slit, posterior anal lip slightly protuberant. Phasmid conspicuous, located less than 1 ABD posterior to anus. Tail smoothly tapering or slightly elongate conical, with pointed terminus.

Supplementary Table S1. The molecular sequences of 115 diplogatrid species compared in the present study. *Rhabditoides inermis* served as outgroup species.

Species	28S (LSU)	18S (SSU)
Rhabditoides inermis (Outgroup species)	EU195981	AF082996
Onthodiplogaster japonica **n. gen., n. sp.**	**LC721118***	
Acrostichus floridensis	LC374587	LC374587
Acrostichus halicti	AB455818	AB455817
Acrostichus megaloptae	AB477074	AB477077
Acrostichus palmarum RGD194	LC374584	LC374584
Acrostichus puri	AB477076	AB477079
Acrostichus rhynchophori	LC374583	LC374583
Acrostichus sp. "femorata"	LC530747	LC530748
Acrostichus ziaelasi	LC530735	LC530736
Allodiplogaster hylobii	KJ877266	KJ877224
Allodiplogaster cf. *lucani*	AB597244	AB597233
Allodiplogaster seani	JX163970	JX163979
Allodiplogaster josephi	EU195999	EU196025
Allodiplogaster sudhausi	KJ877267	KJ877226
Butlerius sp. VS-2014	KJ877247	KJ877204
Cutidiplogaster manati	MT160762	MT160758
Cutidiplogaster sp. “LT”	MT160763	MT160759
Demaniella sp. NKZ367	LC210628	LC210625
Diplogasteriana schneideri	KJ877246	KJ877203
Diplogasteriana sp. RS9000	KJ877245	KJ877202
Diplogasteroides (*Fuchsnema*) *halleri*	KJ877253	KJ877227
Diplogasteroides (*Fuchsnema*) sp. RS5537	KJ877254	KJ877228
Diplogasteroides (*Pseudodiplogaster*) *magnus*	KJ877270	KJ877214
Diplogasteroides (*Pseudodiplogaster*) *nasuensis*	LC0276755	LC027674
Diplogasteroides (*Pseudodiplogaster*) sp. RS5444	KJ877271	KJ877215
Diplogasteroides (*Rhabdontolaimus*) *andrassyi*	AB808723	AB808722
Diplogasteroides (*Rhabdontolaimus*) *asiaticus*	LC027673	LC027672
Diplogasteroides (*Rhabdontolaimus*) *luxuriosae*	LC099974	LC099973
Diplogasteroides nix	LC145090	LC145091
Diplogastrellus gracilis	KJ877249	KJ877216
Diplogastrellus metamasius	EU419762	EU419758
Diplogastrellus (*Metadiplogaster*) sp. RS5608	KJ877248	KJ877205

Diplogastrellus (*Metadiplogaster*) sp. "Tadami"	AB597250	AB597239
Eudiplogasterium levidentus	KJ877258	KJ877206
Fictor platypaillata	-	MW621342
Fictor stercorarius	KJ877282	KJ877235
Fictor sp. RS9001	KJ877280	KJ877233
Fictor sp. RS9002	KJ877281	KJ877234
Koerneria cf. *luziae*	AB597243	AB597232
Koerneria sp. RS9004	KJ877283	KJ877239
Leptojacobus dorci	KJ877277	KF924399
Levipalatum texanum	KJ877257	KJ877221
Mehdinema alii	KJ877285	KJ877213
Micoletzkya buetschlii	KJ877252	JX163973
Micoletzkya calligraphi	KJ531092	KJ531036
Micoletzkya hylurginophila	KJ531102	KJ531046
Micoletzkya inedia	KJ531104	KJ531048
Micoletzkya japonica	JX163967	JX163976
Micoletzkya masseyi	JX163968	JX163977
Micoletzkya palliati	JX163965	JX163974
Micoletzkya sexdentati	KJ531094	KJ531038
Mononchoides compositicola	-	GU943511
Mononchoides kanzakii	MW763063	MW649133
Mononchoides macrospiculum	LN827617	LN827618
Mononchoides striatus	-	AY593924
Mononchoides sp. RS5441	KJ877262	KJ877210
Mononchoides sp. RS9007	KJ877263	KJ877209
Mononchoides sp. RS9008	KJ877264	KJ877211
Mononchoides sp. NK2017	LC210629	LC210626
Neodiplogaster acaloleptae	LC107878	LC107877
Neodiplogaster crenatae	AB326309	AB326310
Neodiplogaster sp. RGD904	AB478641	AB478640
Neodiplogaster sp. RS9009	KJ877265	KJ877212
Neodiplogaster unguispiculata	MH048998	MH049001
Neodiplogaster unguispiculata	MH048996	MH048999
Neodiplogaster unguispiculata	MH048997	MH049000
Oigolaimella attenuata	KJ877276	KJ877219
Oigolaimella sp. RS9010	KJ877275	KJ877218

Oigolaimella sp. RGD844	AB478631	AB478630
Oigolaimella sp. RGD884	AB478633	AB478632
Parapristionchus giblindavisi	JX163972	JX163981
Parasitodiplogaster citrinema	AY840555	AB901285
Parasitodiplogaster maxinema	AB810253	AB901283
Parasitodiplogaster nymphanema	LC109318	LC109317
Parasitodiplogaster obtusinema	LC101737	LC101736
Paroigolaimella micrura	KJ877259	KJ877207
Paroigolaimella stresemanni	KJ877261	KJ877230
Pristionchus aerivorus	KJ705000	KJ704996
Pristionchus americanus	KJ704999	KJ704995
Pristionchus arcanus	KT188878	KT188848
Pristionchus bucculentus	AB852582	AB852581
Pristionchus entomophagus	KT188873	KT188843
Pristionchus exspectatus	KT188879	KT188849
Pristionchus fissidentatus	KJ877273	KT188855
Pristionchus japonicus	KT188880	KT188850
Pristionchus lheritieri	KT188876	KT188846
Pristionchus marianneae	KT188866	KT188836
Pristionchus maupasi	LC011449	LC011448
Pristionchus pacificus	EU195982	U81584
Pristionchus racemosae	KT188888	KT188859
Pristionchus sycomori	KT188886	KT188857
Pristionchus triformis	KT188884	KT188854
Pristionchus uniformis	KJ877272	KJ877236
Pseudodiplogasteroides cf. *compositus*	AB597248	AB597237
Pseudodiplogasteroides sp. SB257	KJ877250	KJ877217
Pseudodiplogasteroides sp. 'Luc8'	AB597249	AB597238
Rhabditidoides aegus	AB597251	AB597240
Rhabditidoides humicolus	AB440322	LC095813
Rhabditidoides sp. RS5443	KJ877251	KJ877229
Rhabditolaimus anoplophorae	AB849949	AB849946
Rhabditolaimus leuckarti	JQ005870	JQ005865
Rhabditolaimus sp. RS5442	KJ877255	KJ877220
Rhabditolaimus sp. RS5414	JQ005871	JQ005866
Rhabditolaimus sp. RSA134	JQ005872	JQ005867

Rhabditolaimus sp. "*Episcapha*"	AB849950	AB849947
Rhabditolaimus sp. "*Euwallacea*"	AB849951	AB849948
Sachsia zurstrasseni	KJ877260	KJ877208
Sudhausia aristotokia	KJ877278	KJ877231
Sudhausia crassa	KJ877279	KJ877232
Sudhausia floridensis	LC214842	LC214841
Teratodiplogaster fignewmani	AB440311	AB440308
Teratodiplogaster sp. 1 VS-2014	KJ877268	KJ877225
Teratodiplogaster sp. 2 VS-2014	KJ877269	KJ877223
Teratodiplogaster variegatae	LC004468	LC004467
Tylopharynx foetidus	-	EU306343
Diplogastridae sp. “ST”	MT160764	MT160760

* Long sequence including near full SSU, ITS and D1-D4 regions of LSU

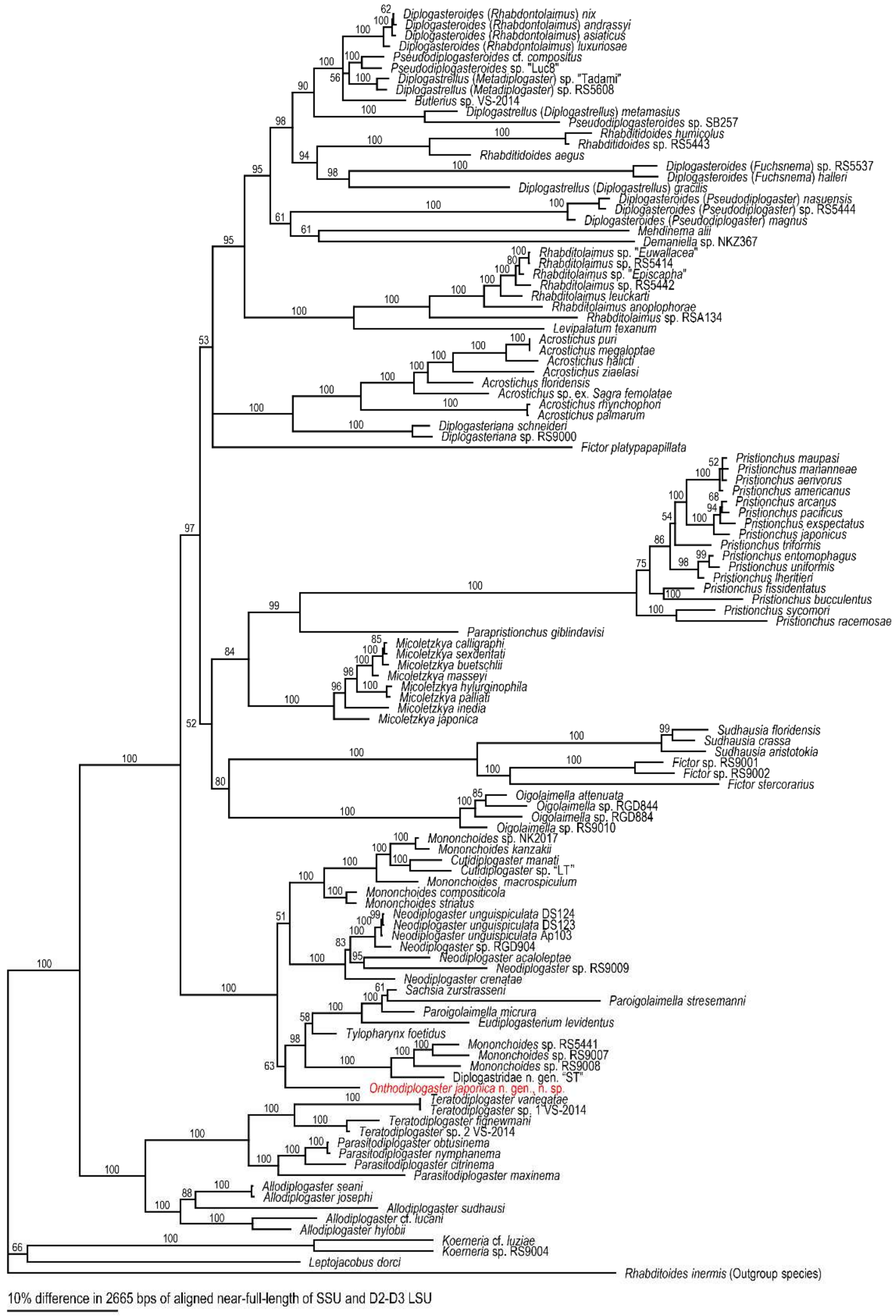

Supplementary Figure S1. Molecular phylogenetic relationships among diplogastrid nematodes showing all compared species.

The Bayesian tree inferred from near full length of SSU and D2-D3 LSU of ribosomal RNA genes. The GTR+G+I model was applied to both loci, and the parameters are as

follows: AIC = 50457.865; lnL= - 24991.621; freqA = 0.25, freqC = 0.21, freqG = 0.27, freqT = 0.27; R(a) = 0.93, R(b) = 2.55, R(c) = 2.11, R(d) = 0.91, R(e) = 3.75, R(f) = 1.00; Pinva = 0.39; Shape = 0.58 for SSU, and AIC = 54594.498; lnL = -27059.552; freqA = 0.21, freqC = 0.22, freqG = 0.32, freqT = 0.25; R(a) = 0.46, R(b) = 1.69, R(c) = 0.90, R(d) = 0.43, R(e) = 3.42, R(f) = 1.00; Pinva = 0.21; Shape = 1.00 for D2-D3 LSU. Posterior probability (PP) values exceeding 50% are given on appropriate clades.

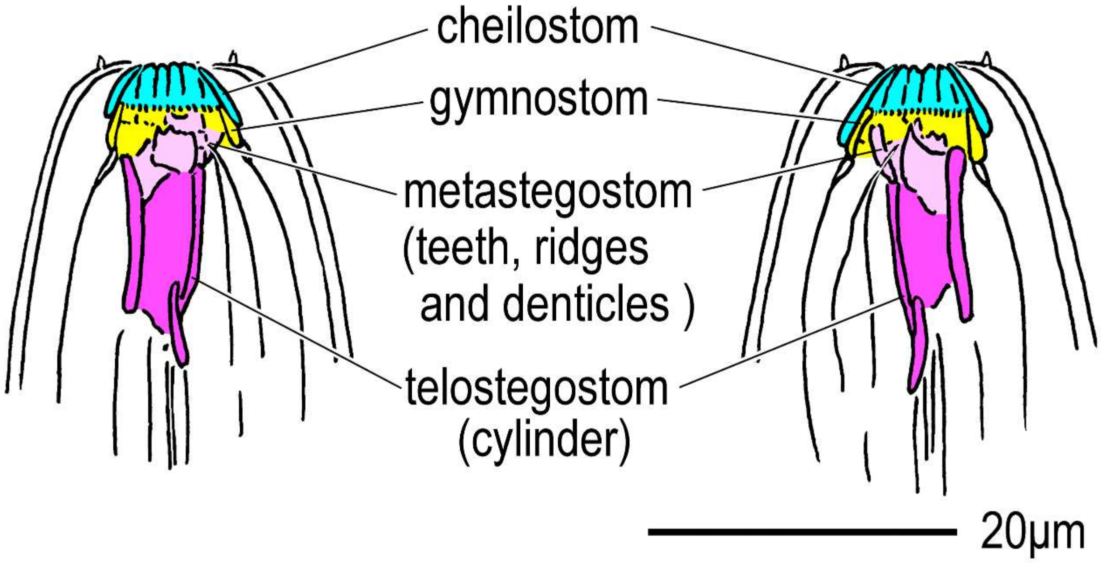

Supplementary Figure S2. Stomatal elements of *Onthodiplogaster japonica* n. gen., n. sp.

Modified after Fig. 2f and 2g, left and right lateral views of anterior part of the predatory female individual of *O. japonica* n. gen., n. sp. Stoma is separated into three sections, cheilostom, gymnostom and stegostom. Further, gymnostom is separated into two subsections, anterior and posterior parts which are associated with anterior and posterior arcade syncitia. While stegostom is separated into three subsections, pro-mesostegostom which is composed by fused prostegostom and metastegostom, metastegostom (teeth, ridges and denticles) and telostegostom (cylinder) from anterior. Pro-mesostegostom is located between gymnostom and metastegostom, but it is not well-developed, and thus, not clearly distinguished by light microscopic observation. Elements mentioned in the text are indicated by different colours.

Supplementary movie S1. *Onthodiplogaster japonica* n. gen., n. sp. large adult female feeding on *Acrobeloides* sp.
Feeding on prey body and leaked body contents moving tooth, stoma and pharynx slowly.

Supplementary movie S2. *Onthodiplogaster japonica* n. gen., n. sp. large adult female feeding on *Acrobeloides* sp.
Feeding on prey body with active movement.

Supplementary movie S3. *Onthodiplogaster japonica* n. gen., n. sp. small adult male feeding on *Botrytis cinerea* mycelia.
Mycelial contents are moving to nematode pharynx.

Supplementary movie S4. *Onthodiplogaster japonica* n. gen., n. sp. juvenile feeding on Mucorales fungal mycelia.
Sometimes show the feeding behavior, but move actively in the agar medium. Nematode is possiblly seeking appropriate food.
